# Sequence-Specific Intramembrane Proteolysis: Identification of a Recognition Motif in Rhomboid Substrates

**DOI:** 10.1016/j.molcel.2009.11.006

**Published:** 2009-12-24

**Authors:** Kvido Strisovsky, Hayley J. Sharpe, Matthew Freeman

**Affiliations:** 1 MRC Laboratory of Molecular Biology, Hills Road, Cambridge CB2 0QH, UK

**Keywords:** PROTEINS

## Abstract

Members of the widespread rhomboid family of intramembrane proteases cleave transmembrane domain (TMD) proteins to regulate processes as diverse as EGF receptor signaling, mitochondrial dynamics, and invasion by apicomplexan parasites. However, lack of information about their substrates means that the biological role of most rhomboids remains obscure. Knowledge of how rhomboids recognize their substrates would illuminate their mechanism and might also allow substrate prediction. Previous work has suggested that rhomboid substrates are specified by helical instability in their TMD. Here we demonstrate that rhomboids instead primarily recognize a specific sequence surrounding the cleavage site. This recognition motif is necessary for substrate cleavage, it determines the cleavage site, and it is more strictly required than TM helix-destabilizing residues. Our work demonstrates that intramembrane proteases can be sequence specific and that genome-wide substrate prediction based on their recognition motifs is feasible.

## Introduction

Despite their relatively recent discovery, rhomboids and other intramembrane proteases are already known to control a wide range of biologically and medically important processes. However, the number of cases in which there is knowledge of their function is tiny compared to the total number of intramembrane proteases known to exist (Wolfe, 2009; Freeman, 2008). Thanks to intensive genetic, biochemical, and structural work, rhomboids are currently the best-understood family of intramembrane proteases. The relatively few that have been characterized regulate processes as diverse as EGF receptor signaling, mitochondrial dynamics, regulation of apoptotic stimuli, and apicomplexan parasite invasion (Freeman, 2008; Urban, 2009). Detailed knowledge of how rhomboids select substrates would further our understanding of their enzymatic mechanism and would also provide a foundation for predicting candidate substrates.

Substrates of intramembrane proteases are generally single-pass transmembrane proteins. Their transmembrane domains (TMDs) adopt a helical conformation to satisfy the hydrogen-bonding requirements of polypeptide backbone polar groups, thus minimizing energetically unfavorable exposure to the hydrophobic core of lipid bilayers (Popot and Engelman, 2000). Such secondary structure elements are generally poor protease substrates and need to be destabilized to become susceptible to proteolysis (Hubbard, 1998; Tyndall et al., 2005). Consistent with this, the substrates of three families of intramembrane proteases, including rhomboids, have been shown to require TM helix-destabilizing residues; these are presumed to facilitate local helix unfolding into an open conformation conducive to cleavage (Ye et al., 2000; Lemberg and Martoglio, 2002; Urban and Freeman, 2003; Akiyama and Maegawa, 2007). Beyond these conformational constraints, no sequence conservation in rhomboid substrates has been reported, although an artificial substrate based on the second TMD of the *E. coli* lactose permease (LacYTM2) was cleaved more efficiently by the *E. coli* GlpG rhomboid when the P1 and P1′ residues immediately flanking the scissile bond (Schechter and Berger, 1967) were small and negatively charged, respectively (Akiyama and Maegawa, 2007).

The ability to predict their substrates would accelerate discovery of the biological role of rhomboids, and understanding substrate determination could provide a framework for such predictions. Initial predictive attempts were based on the helix-destabilizing requirements found in the TMD of *Drosophila* Spitz, the natural substrate of *Drosophila* Rhomboid-1 (Urban and Freeman, 2003; Lohi et al., 2004). This had limited success: a manual search of about 50% (about 1200) of the annotated type I membrane proteins from the mouse genome identified 12 candidate substrates, of which only one was cleaved (Lohi et al., 2004). It was clear even then that the conformational rules applied were insufficient to identify all substrates: for example, the TMD of *Drosophila* Gurken, another natural substrate of fly rhomboids, does not contain a Spitz-like sequence.

In a quest for greater mechanistic understanding of intramembrane proteolysis by rhomboids, and to build a foundation for a more efficient method of substrate prediction, we have investigated rhomboid specificity in detail. Site-directed mutagenesis of rhomboid substrates and enzymatic assays with multiple rhomboid proteases in vitro and in vivo has led us to discover that a previously unrecognized sequence motif in rhomboid substrates is a major determinant of cleavage. This recognition sequence is necessary for substrate cleavage, it determines the position of the cleavage site, and it is more strictly required than TM helix-destabilizing residues in substrates. TM helical instability is indeed significant in some substrates but is secondary to the motif we report here. Similar recognition motifs are present in all four rhomboid substrates we tested and are required by several, even evolutionarily distant, rhomboid proteases. Finally, we demonstrate that identification of this recognition motif provides an essential element in moving toward genome-wide substrate prediction.

## Results

### Diverse Bacterial Rhomboids Share Cleavage Site Specificity

**Figure 1:**
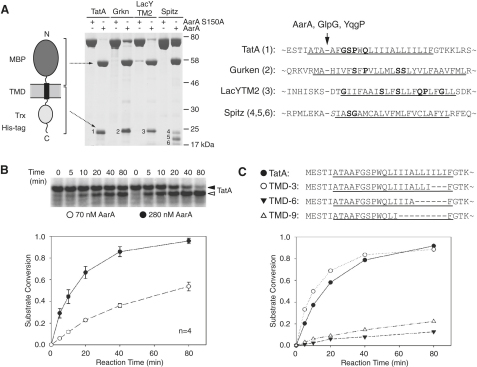
Diverse Bacterial Rhomboids Share Cleavage Site Specificity (A) Fusion proteins containing TMDs of TatA, Gurken, Spitz, and LacYTM2 were cleaved in vitro by bacterial rhomboids AarA (*P. stuartii*), GlpG (*E. coli*), and YqgP (*B. subtilis*). The Coomassie-stained gel shows the C-terminal proteolytic fragments (numbered) that were N-terminally sequenced by automated Edman degradation. The alignment of substrate sequences shows that all three rhomboids cleave each substrate in the same position (indicated by an arrow). TMDs as predicted by the program Phobius (Kall et al., 2004) are underlined, and TM helix-destabilizing residues are shown in bold. (B) Autoradiography shows that the in vitro-translated full-length TatA is cleaved by detergent-solubilized purified AarA in a time- and enzyme-concentration-dependent manner (in this and all subsequent figures, a black arrowhead indicates the substrate and an open arrowhead the cleaved product). Substrate conversion was quantified based on densitometric scanning of the autoradiogram. Data show mean values from four experiments ± standard deviation. (C) Deletions within the TatA TMD show that the intact hydrophobic domain is required for efficient cleavage. AarA concentration was 280 nM, and cleavage rates were evaluated as in (B).

To understand the basis of substrate specificity, we looked for substrate features important for recognition by the enzyme. Using purified components, we determined the cleavage sites in four known model substrates by three different bacterial rhomboid proteases (Figure 1A). The TMDs of *Providencia stuartii* TatA (Stevenson et al., 2007), *E. coli* LacY TMD2 (Maegawa et al., 2005), and *D. melanogaster* Gurken and Spitz (Urban et al., 2002; Lemberg et al., 2005) were engineered into a fusion protein backbone that included a signal peptide and maltose-binding protein (MBP) N-terminal to the TMD, and a thioredoxin (Trx) domain and His tag at the C terminus. We analyzed the cleavage of these four purified recombinant proteins by three bacterial rhomboids: AarA (*P. stuartii)*, GlpG (*E. coli*), and YqgP (*Bacillus subtilis*). These enzymes are quite divergent (http://www.ncbi.nlm.nih.gov/COG/), and they differ in their predicted topology (six TMDs for GlpG, and seven TMDs for AarA and YqgP) and their extramembrane domains. Nonetheless, N-terminal sequencing of the cleavage products showed that all three rhomboids cleaved each substrate at the same position at or very near to the top of the TMD (Figure 1A). Importantly, the insertion into a fusion protein did not affect the site of cleavage of TatA by AarA, LacYTM2 by GlpG, and Gurken by YqgP, which have been previously determined in different contexts and by different methods (Stevenson et al., 2007; Maegawa et al., 2005; Lemberg et al., 2005). There are no obvious sequence similarities among the substrates to explain the invariant cleavage site by evolutionarily diverse rhomboids, although they do all contain TM helix-destabilizing residues (Figure 1A, highlighted in bold). However, since the position of these residues relative to the cleavage site and the TMD is variable, it is difficult to rationalize how they could define the site of cleavage (Ha, 2009).

### TatA as a Model Substrate

To investigate more rigorously whether there are sequence-specific substrate determinants beyond TM helix instability, we focused on a single enzyme and its substrate. We used the *P. stuartii* rhomboid AarA and its physiological substrate TatA (Stevenson et al., 2007), which allowed us to determine substrate cleavage rates and sites both in vitro and in vivo. In vitro-translated L-[^35^S]-Met-labeled TatA was cleaved by AarA in a time- and enzyme-concentration-dependent manner (Figure 1B). A series of deletions within the TMD demonstrated that even when the enzyme and substrate are solubilized in detergent, cleavage rate depends on the integrity of the hydrophobic part of the TMD (Figure 1C), supporting the idea that the in vitro reaction is a valid model of the reaction in vivo, which occurs within a lipid bilayer.

### Role of Transmembrane Helix-Destabilizing Residues in Substrates

**Figure 2:**
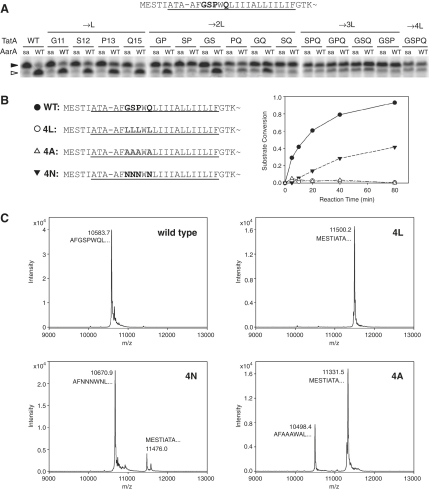
Transmembrane Helix-Destabilizing Residues Are Required for Cleavage of TatA (A) TM helix-destabilizing residues G11, S12, P13, and Q15 in TatA were mutated into leucine in all combinations. The in vitro translated and radiolabeled mutant substrates were assayed for cleavage efficiency using 280 nM AarA and 40 min reaction time to ensure appropriate sensitivity (see Figure 1B). At least two TM helix destabilizers are required for efficient cleavage. WT, wild-type enzyme; SA, catalytic serine-to-alanine mutant. (B) The requirement is specific for residues with low TM helical propensity: substitution of the four TM helix destabilizers by other TM helix-stabilizing residues (alanine) completely blocks cleavage in vitro, whereas other TM helix-destabilizing residues (asparagine) allow substantial cleavage. The in vitro-translated radiolabeled proteins were cleaved by 280 nM AarA. Substrate conversion values were derived from the SDS PAGE gel autoradiogram. (C) The same trend as in (B) is observed in biological membranes. TatA and its mutants were overexpressed in wild-type *P. stuartii* expressing endogenous AarA. Each protein was purified from the membrane fraction through its C-terminal His tag and analyzed by MALDI mass spectrometry. The spectra show the relative proportion of full-length and AarA cleaved forms in each case. The N termini of individual species were inferred from their molecular mass, as indicated. The experimental masses of the uncleaved full-length proteins were consistent with the presence of N-terminal formylmethionine.

We examined in more detail the role of TM helix-destabilizing residues (Engelman et al., 1986; Li and Deber, 1994; Wimley and White, 1996; Hessa et al., 2005) in TatA. As shown in Figure 2A, there are four such residues within its predicted TMD (G11, S12, P13, and Q15), all localized near the rhomboid cleavage site. We substituted them in all combinations with leucine, a TM helix-stabilizing residue, and compared cleavage efficiency (Figure 2A). At least two TM helix-destabilizing residues were required for efficient TatA cleavage. This appears to depend on their TM helical propensity rather than other properties, since other TM helix-stabilizing residues (e.g., alanine) also had a strong inhibitory effect, while residues with poor TM-helical propensity (e.g., asparagine) allowed cleavage, irrespective of their side-chain sizes (leucine and asparagine have larger side chains than glycine, serine, and alanine [Pontius et al., 1996]), both in vitro (Figure 2B) and in biological membranes (Figure 2C). These experiments confirmed that TM helix-destabilizing residues are important for TatA cleavage by AarA but did not address if and how they determine the cleavage site.

### Factors Determining Cleavage Site Position

**Figure 3:**
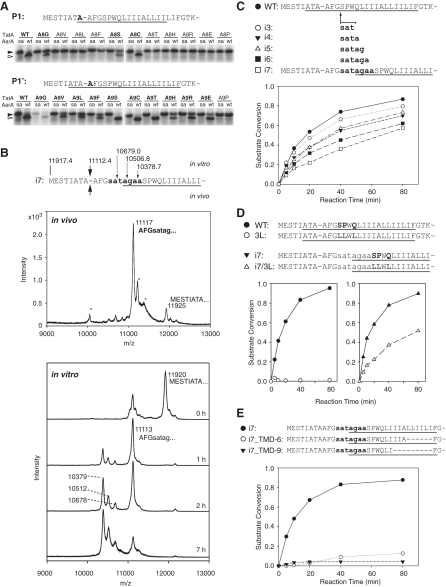
Factors Determining Cleavage Site Position (A) Preferences at P1 and P1′ positions in TatA. A8 and A9 were mutated individually into amino acids whose side chains represent a range of physicochemical properties and analyzed for cleavage by AarA. The P1 position is much more constrained than P1′: tolerated mutations are highlighted in boldface. Enzyme concentration was 450 nM and reaction time 40 min. WT, wild-type; SA, catalytic serine-to-alanine mutant. (B) A linker containing susceptible P1-P1′ pairs was inserted between G11 and S12 of TatA to generate the i7 mutant. As indicated by the large arrows, it is cleaved by AarA in vivo only at the original A8-A9 site. Cleavage also occurs at the same site in vitro, and when overdigested, less-efficient secondary cleavages occur in the linker region (small arrows). The theoretical masses of the C-terminal fragments resulting from i7 cleavage at indicated sites are annotated above its sequence. Upper graph, MALDI mass spectrum of the in vivo-processed i7 mutant that had been expressed in wild-type *P. stuartii* and isolated from the membrane fraction. Experimental masses of [M+H] ions and corresponding N termini are indicated. The N-terminal sequence determined by Edman degradation is highlighted in bold. Masses of the minor peaks marked with asterisks could not be matched to any cleavage product, and their identity was not established. Lower graph, MALDI mass spectra of an in vitro cleavage reaction time course of the i7 mutant that had been expressed in *E. coli* ΔglpG and isolated from the membrane fraction. AarA concentration was 11.2 μM, and recombinant i7 was at 20 μM. (C) Cleavage rate of linker insertion mutants in vitro is negatively proportional to linker length. AarA was at 280 nM. (D) TM helix-destabilizing residues are less important when cleavage occurs outside the bilayer. Substitution of S12, P13, and Q14 by leucine completely blocks cleavage in vitro in the context of otherwise wild-type TatA, but not in the context of the i7 mutant. To ensure comparable cleavage rates between the pairs, AarA concentration was 280 nM for WT and 3L, and 840 nM for i7 and i7/3L. (E) Cleavage rate comparison of TMD deletion variants of the i7 linker insertion mutant showed that even when cleavage occurs outside the TMD, the hydrophobic part of i7 TMD is still required. AarA concentration was 840 nM. In (C)–(E), within each experiment the in vitro-translated radiolabeled substrate variants were equimolar as judged by equal intensity of their bands on the autoradiograms, and the cleavage rates were quantitated as in Figure 1B.

The P1 and P1′ residues that flank the scissile bond are often the most critical for classical serine protease recognition (Hedstrom, 2002; Perona and Craik, 1995) and have been shown to influence cleavage efficiency of a model substrate by the *E. coli* rhomboid GlpG (Akiyama and Maegawa, 2007). We examined the effect of mutating the TatA P1 and P1′ residues individually. As shown in Figure 3A, P1 only tolerated amino acids with a small side chain, such as alanine, cysteine, serine, and glycine. By contrast, the P1′ position was significantly less restricted and could accommodate nearly any amino acid tested except proline, although alanine, cysteine, serine, glycine, threonine, and glutamate were preferred. These TatA preferences were similar but not identical to the constraints found previously for LacYTM2 (Akiyama and Maegawa, 2007). Notably, however, the P1 and P1′ preferences we observed could not explain the unique cleavage site in TatA, since there are four other nearby positions that also fulfil these requirements (S3-T4, A6-T7, A9-F10, and G11-S12) but which were not cleaved. We therefore hypothesized that the distance of a suitable P1-P1′ pair from the TMD or the TM helix-destabilizing residues might direct cleavage site selection.

To test this idea, we introduced an amino acid linker of varying length designed to contain several susceptible P1-P1′ pairs, between G11 and S12 (Figures 3B and 3C). This is predicted to displace the original cleavage site N terminally into the interface or juxtamembrane region, thus separating it from the TM helix-destabilizing region and the hydrophobic TMD core. To our surprise, cleavage still occurred only in the original A8-A9 site both in vitro and in vivo (Figure 3B). Only after overdigestion in vitro could we observe secondary cleavages in the linker, always at the predicted susceptible positions (Figure 3B). We did note that the longer the inserted sequence, the slower the rate of proteolysis (Figure 3C), indicating that there is a rate penalty to be paid as the cleavage site is moved away from the membrane.

Are TM helix-destabilizing residues still required when cleavage occurs outside the membrane? Replacement of S12, P13, and Q15 by leucines completely blocked cleavage in TatA but only moderately inhibited cleavage of its longest linker insertion mutant both in vitro (Figure 3D) and in biological membranes (see Figure S1 available online), implying that if the cleavage site is outside the membrane, TM helical instability is less important. Despite this, the presence of a hydrophobic TMD is still required: deletions of six and more residues from the hydrophobic TMD core of the linker insertion mutant severely inhibited its cleavage by AarA (Figure 3E), just as they did in wild-type TatA (Figure 1C).

### A Primary Recognition Motif in TatA

**Figure 4:**
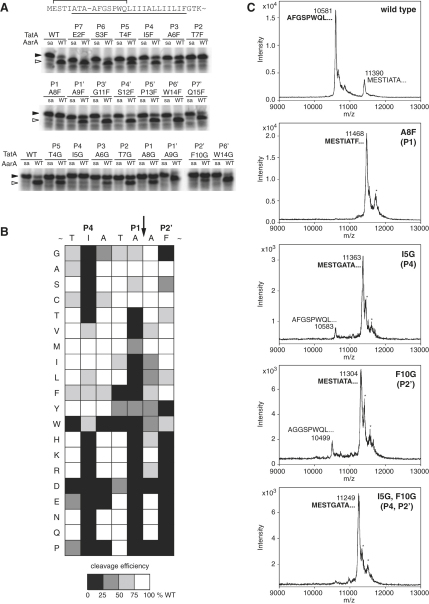
TatA Cleavage Site Is Determined by a Three Amino Acid Recognition Motif (A) Importance of each residue within the TatA cleavage site region, encompassing P7–P7′, was examined by phenylalanine and glycine scanning mutagenesis. Enzyme concentration was 280 nM and reaction time 40 min (see Figure 1B). (B) The P5–P2′ region was further scrutinized by comprehensive positional scanning mutagenesis. The effects of the mutations were graded into four levels based on the mutant substrate conversion at the end of a 40 min reaction in comparison to the wild-type TatA. Enzyme concentration was 280 nM. Notably, the P1, P4, and P2′ positions in TatA are the most sensitive to mutations. A vertical arrow marks the site of cleavage by AarA. (C) Mutations in P4, P1, and P2′ of the TatA recognition motif that inhibit cleavage in vitro have equally strong inhibitory effect in biological membranes in vivo. TatA mutants were overexpressed in *P. stuartii*, isolated from membrane fraction, and N-terminally sequenced. MALDI mass spectra show relative proportions of full-length versus cleaved forms in each case with experimental molecular weights. The N termini corresponding to individual peak masses are indicated; those determined by Edman degradation are shown in bold. Minor peaks marked with asterisks could not correspond to any TatA cleavage product, since their mass was larger than that of full-length TatA; their identity was not established.

The results above suggested the existence of an unrecognized element that defines the TatA cleavage site. We therefore performed a mutagenesis scan of 14 residues surrounding the TatA cleavage site (E2-Q15; i.e., P7–P7′ in standard protease substrate nomenclature) (Figure 4A). Mutations into phenylalanine introduced a bulky side chain that is not accepted in P1, whereas mutations into glycine simulated side-chain ablation of the original residue. The results of the in vitro activity assays suggested that residues T4–F10 (i.e., P5–P2′) are most sensitive to substitutions (Figure 4A). We therefore comprehensively mutagenized each individual position along the whole P5–P2′ region (Figure 4B). It turns out that three positions are particularly sensitive to mutations: P1, already identified as affecting cleavage rate (Figure 3A); P4; and P2′ (Figure 4B). Whereas P1 tolerates only amino acids with a small side chain, P4 requires large and hydrophobic residues, and P2′ prefers hydrophobic side chains irrespective of their size. All other positions between P5 and P2′ can tolerate a variety of amino acids, although tryptophan, proline, and aspartate are deleterious in most of them (Figure 4B). As a confirmation of the importance of the P4, P1, and P2′ positions in determining TatA cleavage, we also substituted all other residues from P7 to P2 simultaneously into alanines and analyzed cleavage of this mutant in vitro. It was cleaved in the same site (A8-A9) and with a similar kinetics as the wild-type TatA (Figure S2), confirming that P4, P1, and P2′ are sufficient to define the cleavage site.

We tested these conclusions in vivo and found the same rules to apply. Overexpression of the wild-type TatA in *P. stuartii* results in its virtually complete cleavage by AarA. By contrast, cleavage of TatA variants mutated in the critical positions, P1 (A8F), P4 (I5G), P2′ (F10G), and P4/P2′ (I5G/F10G), was abrogated, as determined by N-terminal sequencing and mass spectrometry (Figure 4C).

**Figure 5:**
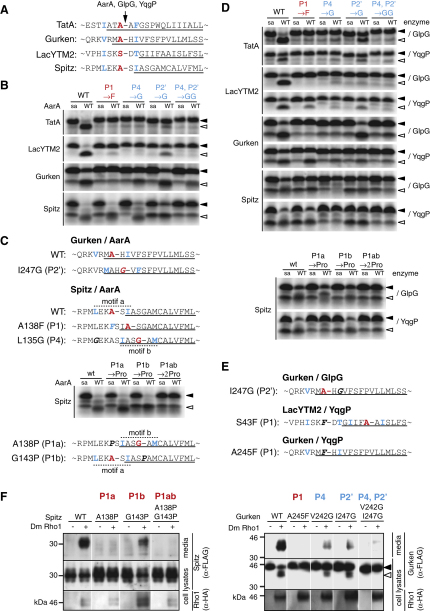
The TatA-like Recognition Motif in Substrates Is Required by Rhomboids from Evolutionarily Distant Species (A) The recognition motif occurs in all four substrates examined. Protein fragments are aligned by rhomboid cleavage site, and the crucial motif residues are highlighted: P1 in red, P4 and P2′ in blue. (B) Recognition motifs in TatA, LacYTM2, Gurken, and Spitz are required by AarA, and they can be disabled by point mutations. The substrates were in vitro translated and radiolabeled; the enzyme was used at 280, 140, 1120, and 224 nM, respectively; and reaction time was 40 min. Note that due to second translation initiation at an internal methionine residue, Spitz and Gurken may contain a weak band of similar mobility as the rhomboid cleavage product. WT, wild-type; SA, catalytic serine-to-alanine mutant. (C) Analysis of cleavage sites in Gurken P2′ and Spitz mutants by N-terminal sequencing and mass spectrometry. The I247G mutation in the P2′ of Gurken and A138F mutation in P1 of Spitz have created new recognition motifs (color coded; the introduced mutations in bold and italicized). Spitz contains a secondary recognition motif (b) that is used when the primary one (a) is knocked out by L135G mutation. Mutations of the P1a and P1b positions of Spitz into proline do not block cleavage individually, but they do so in combination. (D) Bacterial rhomboids GlpG and YqgP require identical recognition motifs in the same set of substrates. Purified GlpG was used at 0.8, 3.2, 6.4, and 0.8 μM, and detergent-solubilized *E. coli* membranes containing YqgP were used at 0.8, 0.2, 0.8, and 1.6 μg/μL for TatA, LacYTM2, Gurken, and Spitz, respectively. (E) Cleavage site analysis by N-terminal sequencing. GlpG is able to cleave a suboptimal version of the recognition motif in Gurken I247G with glycine in P2′. YqgP recognizes a secondary but completely stereotypic recognition motif in LacYTM2 P1 serine-to-phenylalanine mutant, but it can also cleave LacYTM2 and Gurken to some extent even with a phenylalanine in P1 position. (F) Western blots of cell-based cleavage assays showing that *Drosophila* Rhomboid-1 recognizes the same motifs in Spitz and Gurken that are required by bacterial rhomboids. Recognition motif mutations strongly inhibit cleavage of Gurken in cells and secretion of Gurken and Spitz into the media. Superfluous lanes have been cropped out from each gel for clarity (indicated by white lines).

These data reveal a primary structure determinant that directs cleavage of an intramembrane protease substrate. Strikingly, in all four rhomboid substrates that we had earlier examined (Figure 1A), the corresponding P4, P1, and P2′ positions are occupied by residues that conform to the requirements in TatA (compare Figure 5A with Figure 4B). Since these substrates are diverse, including both prokaryotic and eukaryotic proteins, this suggested that the motif reported here might be relevant to evolutionarily distant rhomboids.

### The Recognition Motif Is Functionally Conserved in Multiple Substrates

We examined whether the similar motif observed in LacYTM2, Gurken, and Spitz was essential by measuring the effect on cleavage efficiency in vitro of mutating each P1 residue into phenylalanine and each P4 and P2′ residue into glycine (which were not accepted in TatA at these positions) (Figure 5). Cleavage by AarA was blocked or severely inhibited in all cases, apart from three apparent exceptions (Figure 5B). The P2′ mutant of Gurken (I247G) and P1 and P4 mutants of Spitz (A138F and L135G, respectively) were cleaved at almost wild-type levels, although their cleavage products had different electrophoretic mobility. To examine the products in more detail, we introduced these three mutations into the corresponding chimeric MBP/Trx fusion proteins (Figure 1A) and determined cleavage sites in vitro using N-terminal sequencing and mass spectrometry. Reassuringly, we discovered that, in each case, the mutation introduced a new and stereotypical recognition motif (the I247G mutant of Gurken) or uncovered a normally silent motif (denoted “b” in Spitz), which defined an alternative site of cleavage (Figure 5C, Supplemental Results).

### The Recognition Motif Is Required by Divergent Rhomboid Proteases

The results above show that a conserved recognition motif determines the cleavage of diverse substrates by the *P. stuartii* rhomboid, AarA. Do other rhomboids also recognize the same motif? We tested in vitro our panel of substrate mutants against two evolutionarily distant bacterial rhomboids, GlpG and YqgP. Despite their divergence, both GlpG and YqgP were sensitive to mutations in the recognition motifs in all four tested substrates (Figure 5D), indicating that they recognize the same substrate motif as AarA. The few minor differences in relative sensitivity that we observed (Figure 5E, Supplemental Results) suggest that the amino acid preferences of GlpG and YqgP might be influenced by the recognition motif sequence context.

To test if the recognition motif we have defined is relevant to eukaryotic rhomboids, we examined the sensitivity of *Drosophila* Rhomboid-1 to mutations in the recognition motifs of Gurken and Spitz, its natural substrates. Cell culture assays demonstrate that the P1 and double P4/P2′ mutations in Gurken block its cleavage and abrogate secretion (Figure 5F). Spitz, as shown in Figure 5C and described in the Supplemental Data, contains a secondary recognition motif (b) that was used by AarA when the primary (a) site was disabled by a mutation. The (a) motif in Spitz seems to be the dominant site of cleavage also by *Drosophila* Rhomboid-1 in cells, because while both P1a (A138P) and P1a/P1b (A138P/G143P) mutations abrogate Spitz secretion, the single P1b (G143P) mutation has a weaker effect (Figure 5F).

To summarize these results, we have demonstrated that several diverse rhomboid substrates contain a recognition motif which is necessary for cleavage and which unambiguously determines the scissile bond. This recognition motif is required by evolutionarily distant rhomboid proteases, including those with different transmembrane topologies, although we detect some subtle differences in the importance of the different positions in the motif. A practical consequence of these results is that they suggest a common strategy to generate uncleavable substrate mutants, which have great value when investigating the biological role of rhomboids. For example, conversion of the predicted P1 residue into a proline that is prohibited both in P1 and also in most other positions along the recognition motif (Figure 4B) would minimize the danger of creating new recognition motifs (Figure 5C).

### Substrate Prediction Based on the Recognition Motif

**Figure 6:**
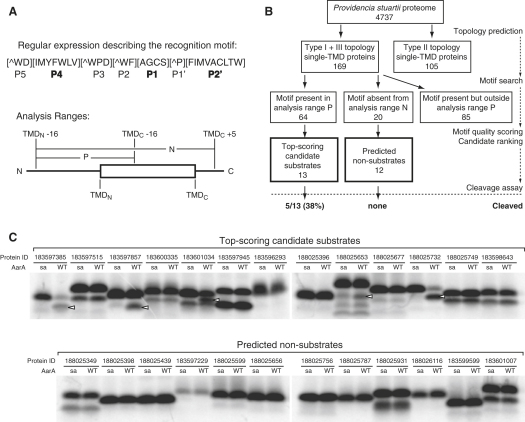
Genome-wide Substrate Predictions Based on the Recognition Motif (A) The regular expression representing the AarA recognition motif derived from the specificity matrix (Figure 4B) that was used to search the *P. stuartii* subproteome of type I and III single-TMD proteins ([ ] matches any single alphabetical character contained within the brackets, and [ˆ ] matches any single alphabetical character not contained in the brackets). Analysis ranges for motif search were limited to “P” for predicted substrates and to “N” for predicted nonsubstrates. (B) Overall analysis workflow. (C) The top-scoring candidate substrates and predicted nonsubstrates were tested for cleavage by AarA in vitro. The protein-encoding fragments were amplified from *P. stuartii* genomic DNA and in vitro translated; AarA concentration in cleavage reactions was 560 nM, and reaction time 40 min. Open arrows denote cleavage products. WT, wild-type; SA, catalytic serine-to-alanine mutant; NCBI IDs for each protein are indicated.

The existence of an essential recognition sequence in substrates could provide an important foundation for predicting candidate substrates from genome sequences. To test this, we analyzed all 4737 predicted proteins in the *P. stuartii* genome and filtered them to identify only those expected to be single spanning transmembrane proteins with a periplasmic N terminus and cytosolic C terminus (i.e., type I or type III, with or without a signal peptide, respectively). Their TMD boundaries were refined with a hydrophobicity-scanning algorithm, and they were searched for a recognition motif (Figure 6A), as defined by the specificity matrix (Figure 4B, for details see the Supplemental Data). Only motifs within a defined sequence window around the N terminus of the TMD were accepted as hits, to reflect our finding that the recognition motif cannot occur too deep within the TMD but can extend at least ten amino acids outside the predicted TMD (analysis range “P,” Figure 6A). The search resulted in 64 type I/III proteins that contained a recognition motif in the analysis range P, 85 proteins that contained the motif outside this analysis range and were thus discarded, and 20 proteins which did not contain any recognition motifs in the analysis range “N” and were thus predicted not to be AarA substrates (Figure 6B). The 64 candidate substrates were then ranked according to their motif quality score based on the specificity matrix, taking into account the identity of amino acids in positions P4, P1, and P2′ (Figure 4B, Supplemental Data).

This procedure yielded a ranked list, in which TatA received top score. To test our predictions, we selected 15 top candidate substrates and 15 predicted nonsubstrates. The corresponding polypeptides were translated in vitro and tested for cleavage by AarA (Figure 6C; for full list of tested TMD sequences, see Figure S3). Strikingly, of those successfully expressed, five out of thirteen top-scoring candidate substrates (38%) were cleaved, as opposed to none of the predicted nonsubstrates (Figure 6C). This suggests that the recognition motif we report provides a useful basis for rhomboid substrate prediction.

## Discussion

The work we report here allows us to infer some important principles of rhomboid substrate recognition. We find that, contrary to previous suggestions, there is a widespread sequence motif that determines the site where diverse rhomboids cleave their substrates: they require a small residue in P1 position and hydrophobic and preferably large residues in P4 and P2′ positions. Previous reports have focused on the conformational requirement for helix-destabilizing residues, missing the existence of a specific primary recognition sequence that determines the site of cleavage. We find that helical instability is indeed required for those substrates in which the cleavage site is within or very near the TMD, but is dispensable when cleavage occurs well outside the membrane.

### How Universal Is the Recognition Motif?

The motif we have discovered directs cleavage by a diverse set of rhomboids and is functionally important in several prokaryotic and eukaryotic substrates; we also find it in previously described rhomboid substrates (see the Supplemental Data). Our data are consistent with a previously reported observation that small residues were favored in P1 position of an artificial rhomboid substrate cleaved by GlpG (Akiyama and Maegawa, 2007). Intriguingly, the motif also explains an earlier genetic result that A245V and A245T mutations in *Drosophila* alleles of Gurken, an EGF receptor ligand that needs cleavage to be active, are equivalent to null mutations: no signaling occurs (Queenan et al., 1999). These mutations are both in the P1 position, and both violate the recognition motif (Figure 4B).

Despite the widespread significance of this motif, we cannot infer that it is universal for all rhomboids. Assuming that the favored residues in the P4, P1, and P2′ positions fit into binding pockets in the active site, cooperative effects between these subsites or the local sequence context might slightly alter the motif preferences, as has been observed previously in other proteases (Ng et al., 2009). It is also plausible that variations in the exact location and identity of the residues lining these pockets may have evolved within the rhomboid family, resulting in different motif preferences among family subgroups. Such evolutionary diversification has been observed, for example, within the well-studied chymotrypsin-like serine proteases that all display structural and sequence similarity (Perona and Craik, 1995; Hedstrom, 2002). Consistent with this idea, mitochondrial rhomboid substrates, whose cleavage sites in vivo have been analytically determined, seem to lack some elements of the P4-P1-P2′ motif that we describe (Herlan et al., 2003; Tatsuta et al., 2007). Similarly, *Plasmodium falciparum* rhomboid 4 (PfROM4) cleaves EBA-175 in vivo at a site that lacks a stereotypical P4 residue (O'Donnell et al., 2006); interestingly, PfROM4 does not cleave Spitz, consistent with the suggestion that it may represent a different specificity class (Baker et al., 2006). Furthermore, there may be additional substrate recruitment mechanisms in some cases: thrombomodulin cleavage by mammalian RHBDL2 depends on a cytoplasmic domain in the substrate (Lohi et al., 2004), and HtrA2 possibly requires an additional protein factor for its presentation to the mitochondrial rhomboid PARL (Chao et al., 2008), although this requirement has recently been challenged (Jeyaraju et al., 2009).

Notwithstanding the possible variations between rhomboid specificities, the most direct message of this work is that the requirement for the recognition motif that we have discovered is conserved among diverse rhomboid enzymes from different species and with different numbers of TMDs. Since all rhomboids are presumably mechanistically related (Lemberg and Freeman, 2007a, 2007b), the recognition motif represents an important and general determinant of cleavage and may provide a framework for all substrate recognition. More broadly, our work provides evidence that an intramembrane protease exploits a primary sequence motif to recognize and direct substrate cleavage.

### Implications for Rhomboid Mechanism of Substrate Recognition

Recent structural and biochemical work on GlpG has led to two models of substrate access: (1) lateral entry of a substrate TMD into the enzyme core between helices 2 and 5 (Baker et al., 2007; Wu et al., 2006), and (2) thinning of the lipid bilayer around the enzyme creating hydrophobic mismatch that facilitates partitioning of the top part of substrate TMD out of the lipid bilayer (Wang et al., 2007; Akiyama and Maegawa, 2007; Maegawa et al., 2007). These two models, while having common elements and not totally incompatible (Ha, 2009; Lemberg and Freeman, 2007a), do represent substantially different views of substrate access—one of the major questions that remains unresolved for all intramembrane proteases.

**Figure 7:**
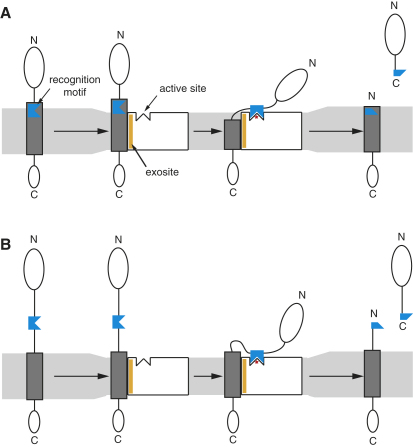
An Updated Model for Rhomboid Substrate Recognition We suggest that rhomboid substrates are defined by two main specificity-conferring elements. A substrate's TMD binds the intramembrane-located “exosite” on the rhomboid enzyme while the recognition motif has affinity to the solvent-exposed rhomboid active site region. Both elements are distinct and separable in a substrate's primary structure. (A) Substrates with a recognition motif located within or near the N terminus of their TMD require the presence of downstream TM helix-destabilizing residues that facilitate local unfolding; this allows the recognition motif access to the rhomboid active site. (B) Substrates with a recognition motif located outside the TMD do not require TM helix-destabilizing residues, since the linker region between the motif and the TMD is sufficient to allow the motif access to the rhomboid active site.

Our results tend to favor the second model, albeit indirectly, and do not demonstrate any need for lateral entry into the core of the enzyme. We propose two stages of substrate recruitment. The requirement for a TMD, even when the cleavage site is outside the membrane, or when it occurs in a detergent-solubilized state, suggests that the initial recognition and recruitment of transmembrane proteins occurs via an intramembrane “exosite” (Fenton et al., 1988; Overall, 2002) on the rhomboid enzyme. Subsequently, the recognition motif is proposed to dock into the active site cleft (Figure 7). The distance between the two elements can vary, and a recognition motif can reside outside the TMD. Indeed, one of our striking observations is that a cleavage site within a TMD can be moved outside of it, implying that the exact position with respect to the membrane is not critical.

Our model (Figure 7) provides a rationalization for the previously observed requirement for TM helix-destabilizing residues in substrate TMDs: if the recognition motif is immediately adjacent to or within the TMD, residues with low TM helical propensity are important. They presumably allow local unfolding of the substrate TM helix, thus facilitating the access and presentation of the recognition motif to the active site cleft. This might also explain why TM helix-destabilizing residues introduced into the TMD relatively distant from the cleavage site seem to facilitate substrate cleavage (Akiyama and Maegawa, 2007; Ha, 2009). However, when the recognition motif resides outside the membrane, TM helix destabilizers are less important because the structural flexibility needed for motif access to the active site is endowed by the sequence between the cleavage site and the TMD. This model might also explain the observation that a type II membrane protein, Star, could be a rhomboid substrate (Tsruya et al., 2007). The “lateral entry” model implies that Star must bind the rhomboid active site in the opposite polypeptide orientation to other known substrates. In contrast, our model predicts that, if the recognition motif in Star is sufficiently far from the TMD, it could still loop back into the active site and bind with the “correct” orientation.

### Substrate Prediction

Since rhomboids are proteases, identifying their substrates is the key step in defining their biological roles. We now identify several factors that in concert define a substrate: presence and quality of the recognition motif, its position relative to the TMD, and the TMD character, including its predicted helical stability. Our analysis of the *P. stuartii* genome demonstrates that the recognition motif alone can already be used to produce lists of candidate rhomboid substrates. Moreover, since this pilot focused only on the recognition motif, and neglected scoring for other known factors, such as distance of the motif from the TMD or presence of TM helix-destabilizing residues, additional improvement of the prediction algorithm should be achievable, raising the realistic possibility of efficient, semiautomated identification of candidate rhomboid substrates. Of course, experimental support will always be needed to validate such predictions. Finally, it will be interesting to discover whether other intramembrane proteases like gamma-secretase and the SPP-like and S2P families also require sequence-specific recognition motifs that can provide a basis for substrate prediction.

## Experimental Procedures

### Constructs and DNA Cloning

Site-directed mutagenesis was done by the QuikChange protocol (Stratagene) or overlap extension PCR (Ho et al., 1989). TatA mutants were constructed in pET21a.TatA.His6 (Stevenson et al., 2007) or in its derivative pKS187, which was generated by extending the TatA construct at its N terminus by an (SG)_4_ tag using PCR. LacYTM2 and its mutants used for the radioactive in vitro assay were created in the background of the Bla-LacYTM2-MBP construct pGW93 (Maegawa et al., 2005), which we generated by PCR assembling its fragment that encoded sequence from RWEPEL of β-galactosidase (Bla) to AVEALSL of MBP and cloning it into pUC19 between EcoRI and HindIII sites to give pKS205. *Drosophila* Spitz and Gurken genes were cloned in pcDNA3.1 with an added N-terminal triple FLAG tag to yield pcDNA.3FLAG-N.Spitz and pcDNA.3FLAG-N.Gurken.

The chimeric substrate backbone used for determination of cleavage sites was created by cloning a DNA fragment encoding Trx domain with a C-terminal His and S tags from pET32a+ (residues MSDKII to the C terminus) between XbaI and HindIII sites of pMALp2E, which had been modified by inserting a triple FLAG tag encoding oligonucleotide in-frame between SacI and AvaI sites, to yield pKS29. Individual chimeric substrates were created by inserting DNA fragments encoding TMDs and the adjacent regions of TatA (NCBI Protein ID ABM10849, residues M1–M50), LacY (AP_000995.1, TM2, residues H39–K74), Gurken (AAA28598, residues Q239–Q288) and Spitz (AAA28894, residues P131–Y181) in-frame between KpnI and XbaI sites of pKS29, to yield pKS273, pKS35, pKS34, and pKS230, respectively.

### Protein Expression and Purification

Bacterial rhomboids AarA, GlpG, and YqgP (NCBI Protein ID AAA61597.1, YP_026220.1, and NP_390367.1, respectively) and their active site mutants AarA.S150A, GlpG.S201A, and YqgP.S288A were overexpressed in *E. coli* BL21(DE3) as full-length, C-terminally His-tagged proteins as described (Lemberg et al., 2005). Recombinant chimeric substrates (MBP-TMD-Trx-His6) and mutant TatA variants were overexpressed in *E. coli* MG1655 ΔglpEGR::kan (gift of Philip N. Rather, Emory University School of Medicine) to prevent possible endogenous rhomboid cleavage. For investigation of in vivo cleavage of TatA mutants, electrocompetent *Providencia stuartii* or its *aarA* mutant (Rather and Orosz, 1994) was cotransformed with pET21a.TatA.His6 and p184.T7 bearing IPTG-inducible T7 RNA polymerase gene (Macinga et al., 1999). Transformants were selected on 300 μg/mL ampicillin and 100 μg/mL chloramphenicol LB plates. To express the recombinant substrates, transformed cultures were grown at 37°C in LB medium, supplemented with appropriate antibiotics to OD_600_ of 0.8, and induced by 0.5 mM IPTG for 2–3 hr. Cellular membranes were isolated and solubilized in 1.5% (w/v) n-dodecyl-β-D-maltoside (DDM, Glycon Biochemicals GmbH, Germany) as described (Lemberg et al., 2005). Detergent-solubilized His-tagged proteins were purified on NiNTA agarose (QIAGEN) as described (Stevenson et al., 2007) except for YqgP, which was used as DDM-solubilized membranes.

### In Vitro Assay of Rhomboid Activity

The radiolabeled protein substrates for rhomboid activity assay were generated by in vitro translation in the presence of L-[^35^S]-Met (EasyTag, Perkin Elmer) using wheat germ extract (Promega) as described (Lemberg and Martoglio, 2003). RNA templates were prepared by in vitro transcription with SP6 RNA polymerase (New England Biolabs) of PCR products generated from constructs pET21a.TatA.His6, pKS205, pcDNA.3FLAG-N.Spitz, and pcDNA.3FLAG-N.Gurken and their point mutant derivatives using specific primers (Lemberg et al., 2005). Translation products corresponded to polypeptide segments E2–G98 for TatA, DERNRQ–LRKTSK for Bla-LacYTM2-MBP fusion, A223–R271 for Gurken, and G114–L161 for Spitz, always preceded by the initiator Met and ending with three additional Met residues at the C terminus. In the case of TatA, to increase the size difference between the substrate and rhomboid cleavage product and thus improve their electrophoretic resolution, the initiator Met was followed by a (GS)_4_ tag (Stevenson et al., 2007), which does not alter the specificity and kinetics of cleavage (data not shown). Alternatively, TatA mutants generated in pKS187 were in vitro transcribed and translated using the *E. coli* T7 S30 Extract System for Circular DNA (Promega).

Cleavage reactions were conducted at 37°C for the indicated time, typically in 20 μl volume of reaction buffer consisting of 50 mM HEPES-NaOH (pH 7.5), 5 mM EDTA, 10% (v/v) glycerol, and 0.05% (w/v) DDM, with 2–4 μl of translation mixture and the indicated amounts of rhomboid. GlpG reactions were supplemented with 0.4 M NaCl to enhance cleavage rate. Reactions were stopped by 10% (w/v) trichloroacetic acid (TCA) and products separated by 10% BisTris-MES SDS PAGE (Invitrogen). Gels were dried and autoradiographed. Substrate conversion of TatA was calculated from the densitometric analysis of the autoradiogram, assuming uniform labeling by L-[^35^S]-Met and correcting for the number of Met residues in substrate and cleavage products.

### Determination of Rhomboid Cleavage Sites

For the analysis of in vitro cleavage sites, purified MBP-TMD-Trx-His6 chimeras or TatA variants were incubated with the indicated rhomboid protease for 2–4 hr at 37°C in the presence of 50 mM HEPES-NaOH (pH 7.5), 5 mM EDTA, 10% (v/v) glycerol, and 0.05% (w/v) DDM. For in vivo cleavage, TatA mutants were expressed in *P. stuartii*, isolated, and purified by DDM-solubilization of cellular membranes and NiNTA chromatography. Proteins were separated on SDS PAGE and electroblotted onto a PVDF membrane (Immobilon-PSQ, Millipore). N-terminal sequence of the C-terminal cleavage products was determined by Edman degradation using a Procise Protein Sequencing System (491 Protein Sequencer, PE Applied Biosystems). Alternatively, MBP-TMD-Trx-His6 cleavage reactions were subjected to microscale NiNTA chromatography, which removed the N-terminal cleavage product. Molecular weights of protein fragments were determined by MALDI-TOF mass spectrometry using α-cyano-4-hydroxycinnamic acid (Figures 3B and 4C) or 2,5-dihydroxyacetophenone (Figure 2C and Figure S1) matrix on a Voyager-DE PRO instrument (Applied Biosystems) operated in linear positive mode, typically taking 450 shots per spectrum. Cleavage site positions were inferred from the known sequences and experimental mass values.

### Cell Culture and Cell-Based Assay of Rhomboid Activity

COS7 cells were maintained in Dulbecco's modified Eagle's medium (DMEM + GlutaMax, GIBCO) supplemented with 10% fetal calf serum. Cells were transfected in 6-well plates by FuGene6 (Roche) using total amount of 1 μg of DNA per each 35 mm well as described (Lee et al., 2001). This included 250 ng of plasmid encoding rhomboid substrate, 25 ng of rhomboid, in case of using Spitz as a substrate also 250 ng of Star-encoding plasmid, and optionally 250 ng of pAAV.EGFP (Stratagene) as a transfection efficiency control. Total DNA amount was adjusted to 1 μg by empty vector pcDNA3.1(+). Eighteen hours posttransfection, medium was exchanged for serum-free DMEM supplemented with 10 μM metalloprotease inhibitor BB-94 (British Biotech) to suppress possible background shedding of rhomboid substrates. After further 27 hr, medium was collected, spun at 16,000 g for 10 min at 4°C to remove cell debris, and the supernatant precipitated by adding TCA to 12% (w/v). After centrifugation and acetone wash, the pellet was dissolved in SDS PAGE sample buffer, and samples were analyzed by SDS PAGE using 10% BisTris-MOPS or 4%–20% Tris-glycine gradient gels (Invitrogen) and by western blotting using mouse monoclonal anti-FLAG M2 antibody conjugated to horseradish peroxidase (HRP) (1:2000, Sigma) or mouse monoclonal anti-HA 16B12 (1:2000, Covance) and goat anti-mouse IgG-HRP conjugate (1:5000, Santa Cruz Biotechnology). HRP activity was detected by enhanced chemiluminescence (GE Healthcare).

## Supplemental Data

Document S1. Supplemental Experimental Procedures, Supplemental Results, Supplemental Discussion, Supplemental References, and Three Figures
