# Supplementary material for: Sequence-Specific Intramembrane Proteolysis: Identification of a Recognition Motif in Rhomboid Substrates
Source: Mol Cell. 2009 Dec 24;36(6-2):1048–59. doi: 10.1016/j.molcel.2009.11.006 (PMC2941825; doi:10.1016/j.molcel.2009.11.006)
Supplement: Document S1. Supplemental Experimental Procedures, Supplemental Results, Supplemental Discussion, Supplemental References, and Three Figures [file mmc1.pdf]

Molecular Cell, Volume *36*

Supplemental Data

Sequence-Specific Intramembrane Proteolysis: Identification of a Recognition Motif in Rhomboid Substrates

Kvido Strisovsky, Hayley J. Sharpe, and Matthew Freeman

Supplemental Experimental Procedures

Rhomboid substrate prediction

The proteome sequences of *P. stuartii* were downloaded from NCBI (Accession number NZ_ABJD00000000, that represents a collection of whole genome shotgun sequences). Putative single pass transmembrane proteins were identified using the combined signal peptide and TMD prediction program Phobius (Kall et al., 2004). A hydrophobicity-scanning algorithm (Sharpe et al., unpublished) was used to refine the transmembrane boundaries of proteins predicted to have type I or type III topology (i.e. periplasmic N-terminus). The approximate edges from Phobius were indented by 4 amino acids at both ends and a window of five residues was used to scan for mean hydrophobicity using the Goldman-Engelman-Steitz hydrophobicity scale (Engelman et al., 1986) which is the most appropriate hydrophobicity scale for amino acids in single pass transmembrane regions (Koehler et al., 2009). Transmembrane domain boundaries were then defined by a window average hydrophobicity of greater than -0.94 kcal/mol or by individual residue hydrophobicity of greater than 8.0 kcal/mol.

To identify proteins that contained the rhomboid recognition motif, the specificity matrix (Fig. 4B) was used to derive a regular expression describing the recognition motif as follows. The P4, P1 and P2’ positions were allowed to be occupied only by amino acids that, in the context of TatA, permitted 51-100% cleavage efficiency in comparison to the wild type (denoted by white and light grey squares in Fig. 4C). All other positions, that is, P5, P3, P2, and P1’, were allowed to be occupied by any amino acids except for those that caused decrease of cleavage efficiency to 0-25% compared to the wild type TatA (black squares in

Fig. 4B), unless they occurred in a known substrate (that is glutamate in P3 of Spitz, and aspartate in P1’ of LacYTM2). Next, the putative type I and III proteins were scanned for the regular expression within a sequence window such that the P2’ position of the motif (Fig.6A) was up to 10 amino acids upstream of the N-terminal TMD boundary and greater than 16 residues upstream of the C-terminal boundary of the TMD (analysis range P). Motif quality in these hits was scored by summing up P4, P1, and P2’ contributions following this arbitrary scheme:

Position P4: I, M, or Y = 4; F or W = 3; V or L = 2

Position P1: AC = 6; GS = 4

Position P2’: FIMVAC = 3; LTW = 2

Putative type I or Type III proteins that did not have any motif in a sequence window defined such that the P2’ position was no more than 10 amino acids upstream of the N-terminal TMD boundary and no more than 5 amino acids downstream of the C-terminal TMD boundary (analysis range N) were predicted not to be AarA substrates. Proteins that contained the motif downstream of analysis range P were excluded from further analysis because their motifs were deemed topologically unlikely to be accessible to rhomboid cleavage when embedded in the lipid bilayer.

For cloning purposes, the corresponding nucleotide sequences were obtained using the tblastn function of BLAST (Altschul et al., 1990) on *P.stuartii* contigs downloaded from The Genome Center at Washington University School of Medicine in St. Louis (http://genome.wustl.edu/pub/organism/Microbes/Human_Gut_Microbiome/Providencia_stuartii/assembly/Providencia_stuartii-2.0.1/output/).

Supplemental Results

Newly generated or unmasked secondary recognition motifs explain the apparent exceptions in motif recognition by AarA.

As described in the main text, the P2’ mutant of Gurken (I247G) and P1 and P4 mutants of Spitz (A138F and L135G, respectively) were unexpectedly cleaved at almost wild type levels by AarA. To examine the products in more detail, we introduced these three mutations

into the corresponding chimeric MBP/Trx fusion proteins (Fig. 1A), and determined cleavage sites by AarA *in vitro* using N-terminal sequencing and mass spectrometry (Fig. 5C).

Specifically, the P2' mutation of Gurken (I247G) led to cleavage between G247 and V248 and generated a new recognition site with M244, G247, and F249 in P4, P1, and P2' positions, respectively. Similarly, the P1 (A138F) mutation in Spitz generated a new recognition motif with the F138 in P4 position resulting in cleavage between A141 and S142. Knocking out the original P4 residue in Spitz by L135G mutation shifted the cleavage site between G143 and A144, revealing a secondary recognition motif, normally cryptic, which contains permissive P1 (G143), P4 (I140) and P2' (M145) residues (Fig. 5C). The double P4/P2' mutant of Spitz (L135G/I140G) is uncleavable because P4 residues from both recognition motifs become disabled. The presence of two juxtaposed recognition motifs in Spitz was also confirmed in another way: individual mutation of the primary (A138P) or secondary (G143P) P1 residues in Spitz allows cleavage within the other, non-mutated motif, while the double mutant (A138P/G143P) is uncleavable (Fig. 5D). Thus, the three exceptions were indeed only apparent; they in fact strongly supported and confirmed the overall functional conservation and significance of the TatA-like recognition motif in a diverse set of substrates.

Minor differences in motif recognition by GlpG and YqgP

As with AarA, there were three substrate mutants that appeared partially to contravene the recognition motif requirement. Gurken P2' mutant I247G was cleaved at wild type levels by GlpG, and LacYTM2 and Gurken P1 mutants (S43F and A245F, respectively) were cleaved with moderate efficiency by YqgP. We therefore introduced these three mutations into the corresponding MBP/Trx fusion proteins and determined the cleavage sites by N-terminal sequencing of the *in vitro* reaction products (Fig. 5E). The P2' mutant of Gurken (I247G) is cleaved by GlpG at its original site indicating that GlpG, while broadly conforming to the substrate motif, has slightly different preferences at the P2' position (which is less tightly constrained than the P4 or P1 positions even for AarA). In addition, YqgP recognises a secondary, normally hidden, recognition site in LacYTM2 that is perfectly stereotypical (Fig. 5E). Although this is the major cleavage site in the P1 mutant of LacYTM2 (S43F), minor cleavage at the mutated original site still occurs. More surprisingly, the P1 mutant of Gurken (A245F) is cleaved at the mutated recognition site with moderate efficiency by YqgP, which is surprising because phenylalanine in the P1 position has proven highly deleterious in other

cases. Overall, these results indicate that subsite preferences of GlpG and YqgP for the critical motif positions may be slightly different from those of AarA or that their preferences may be influenced by the recognition motif sequence context.

Supplemental Discussion

Putative recognition motifs in published rhomboid substrates

Our analysis of rhomboid specificity suggests that those rhomboids that can cleave the model substrates Spitz, Gurken, TatA and LacyTM2 all specificially recognise a sequence motif, that we defined by mutagenic analysis of TatA. This conclusion is consistent with published data on other rhomboids and their substrates. For example, mouse RHBDL2 (which can cleave Spitz) was shown to cleave mouse thrombomodulin in a cell-based assay and the cleavage site position has been mapped approximately between proline 508 and leucine 528 (Lohi et al., 2004). Consistent with this, we find at least one stereotypical recognition motif in this region (putative P1 residues will be displayed in bold red, while P4 and P2' in bold blue, and TMD will be underlined): PP$_{508}$AVG**L**VH**S**G**L**LIG**I**SI**A**S**L**CL$_{528}$VVALLALLCHLRKKQ. In addition, human RHBDL2 cleaves human EphrinB3 roughly between proline 226 and cysteine 250 (Pascall and Brown, 2004) and there is at least one stronger and one weaker putative recognition motif in this range: SMP$_{226}$A**V**AG**A**A**G**GLALLL**L**GV**A**G**A**GGAMC$_{250}$WRRR. It remains to be tested whether and in which of these predicted recognition motifs RHBDL2 cleaves.

It seems that even some rhomboids with apparently distinct substrate specificity might require a stereotypical TatA-like recognition motif. The recently identified *Entamoeba histolytica* rhomboid 1 (EhROM1) was shown to cleave an *E. histolytica* surface lectin EHI_044650 in a cell-based overexpression assay (Baxt et al., 2008). Strikingly, cleavage of EHI_044650 is blocked by a glycine to valine mutation that occurs in a putative P1 position within a potential stereotypical recognition motif: QDVDNTAA**I**AA**G**T**T**VAVVVAVIVVVMVIIAIGIKQTV. This is intriguing since EhROM1 does not cleave Spitz and it was suggested to have different substrate specificity from *D. melanogaster* Rhomboid-1 or bacterial rhomboids (Baxt et al., 2008), but it seems that it might recognise a variant of the TatA-like recognition motif.

Finally, we can reconcile our data on Spitz cleavage sites with those published by others. Baker et al. who used Spitz TMD transplanted into C100-Flag (Urban and Wolfe, 2005), originally a gamma-secretase substrate (Li et al., 2000), detected by mass spectrometry two *in vitro* cleavages by GlpG between (in Spitz numbering) A141-S142 and G143-A144 (Baker et al., 2007). In contrast, here we report cleavage of wild type Spitz sequence by three different bacterial rhomboids and by *Drosophila* Rhomboid-1 primarily between amino acids A138-S139. However, we also identify a secondary recognition motif with a cleavage site between G143-A144, which is employed, albeit less efficiently, when the primary motif is mutated (Fig. 5C). This secondary motif is preserved and indeed cleaved by GlpG in C100Spitz-Flag, presumably because the primary motif had been disrupted by the transplantation of Spitz TMD into the C100-Flag context (C100-Flag in uppercase, Spitz in lowercase with Spitz numbering used, secondary recognition motif highlighted): DVG***S***NKa$_{138}$s**i**as**g**a**v**ggvviatvivitlvmlKKK. Note that the residue that would correspond to a P4 in the primary Spitz motif is a serine (bold italicized) in C100Spitz-Flag, which we found non-permissive in this position. In summary, disruption of the primary recognition motif in C100Spitz-Flag leads to cleavages at normally less favoured sites. This is similar to what we have observed in the i7 linker insertion mutant of TatA: during overdigestion of i7 by AarA *in vitro*, susceptible P1-P1'sites in the linker, lacking stereotypic P4 and P2' residues, can be eventually cleaved, albeit considerably more slowly than the true recognition motif (Fig. 3B, lower graph).

Supplemental References

Altschul, S. F., Gish, W., Miller, W., Myers, E. W., and Lipman, D. J. (1990). Basic local alignment search tool. J. Mol. Biol. *215*, 403-410.

Baker, R. P., Young, K., Feng, L., Shi, Y., and Urban, S. (2007). Enzymatic analysis of a rhomboid intramembrane protease implicates transmembrane helix 5 as the lateral substrate gate. Proc. Natl. Acad. Sci. U. S. A. *104*, 8257-8262.

Baxt, L. A., Baker, R. P., Singh, U., and Urban, S. (2008). An Entamoeba histolytica rhomboid protease with atypical specificity cleaves a surface lectin involved in phagocytosis and immune evasion. Genes Dev. *22*, 1636-1646.

Engelman, D. M., Steitz, T. A., and Goldman, A. (1986). Identifying nonpolar transbilayer helices in amino acid sequences of membrane proteins. Annu. Rev. Biophys. Biophys. Chem. *15*, 321-353.

Kall, L., Krogh, A., and Sonnhammer, E. L. (2004). A combined transmembrane topology and signal peptide prediction method. J. Mol. Biol. *338*, 1027-1036.

Koehler, J., Woetzel, N., Staritzbichler, R., Sanders, C. R., and Meiler, J. (2009). A unified hydrophobicity scale for multispan membrane proteins. Proteins *76*, 13-29.

Li, Y. M., Lai, M. T., Xu, M., Huang, Q., DiMuzio-Mower, J., Sardana, M. K., Shi, X. P., Yin, K. C., Shafer, J. A., and Gardell, S. J. (2000). Presenilin 1 is linked with gamma-secretase activity in the detergent solubilized state. Proc. Natl. Acad. Sci. U. S. A. *97*, 6138-6143.

Lohi, O., Urban, S., and Freeman, M. (2004). Diverse substrate recognition mechanisms for rhomboids; thrombomodulin is cleaved by Mammalian rhomboids. Curr. Biol. *14*, 236-241.

Pascall, J. C., and Brown, K. D. (2004). Intramembrane cleavage of ephrinB3 by the human rhomboid family protease, RHBDL2. Biochem. Biophys. Res. Commun. *317*, 244-252.

Urban, S., and Wolfe, M. S. (2005). Reconstitution of intramembrane proteolysis in vitro reveals that pure rhomboid is sufficient for catalysis and specificity. Proc. Natl. Acad. Sci. U. S. A. *102*, 1883-1888.

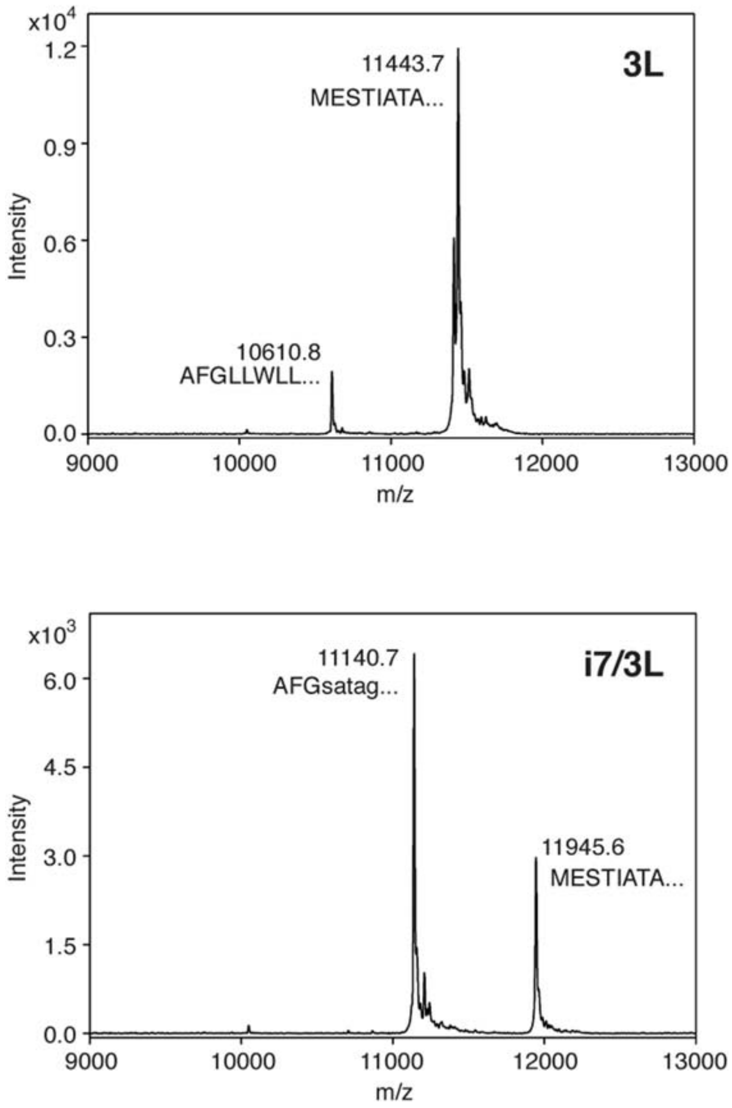

Figure S1. TM helix destabilizing residues are less important when cleavage occurs outside the membrane: *in vivo* confirmation

TatA mutants were overexpressed in wild type *P.stuartii.* Each protein was purified from the membrane fraction by NiNTA chromatography via its C-terminal His-tag, and analysed by MALDI mass spectrometry. The N-termini of individual species were inferred from their molecular mass, as indicated. The experimental masses of the uncleaved full-length proteins were consistent with the possible presence of N-terminal formyl-methionine.

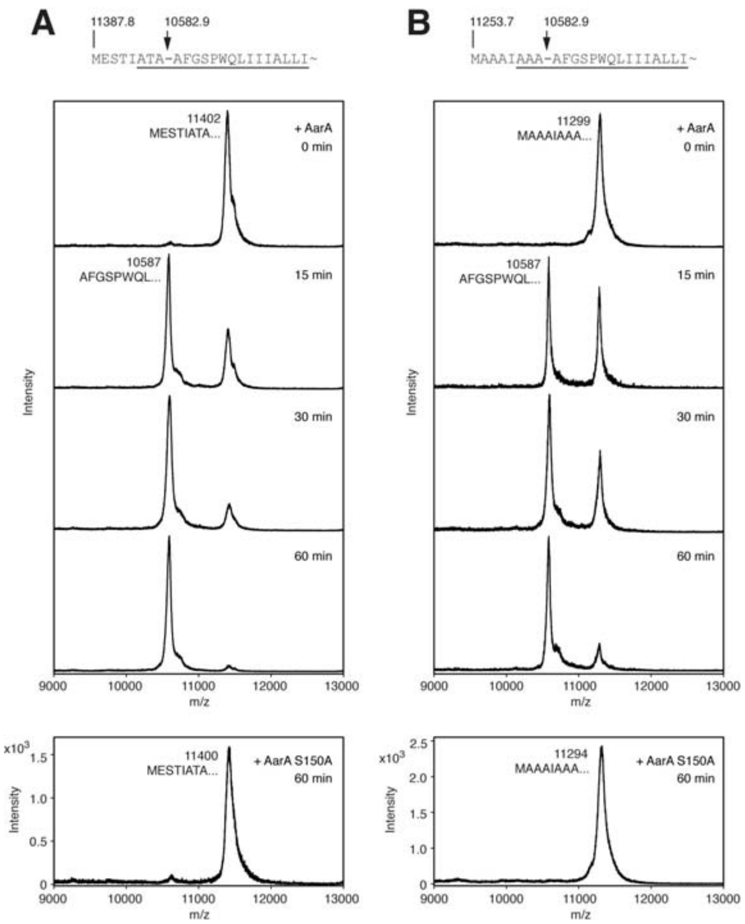

Figure S2. The recognition motif within an oligoalanine context in a mutant TatA is sufficient to define precisely the cleavage site and confer almost wild type cleavage efficiency

Full-length wild type TatA and its E2A/S3A/T4A/T7A mutant were cleaved *in vitro* by purified AarA. Substrates (600 µg/mL) were incubated with AarA (25 µg/mL) at 37ºC in the presence of 20 mM HEPES pH 7.5 and 0.05% (w/v) DDM. At indicated time-points the substrate and product were analysed by MALDI mass spectrometry. As a control, both substrates were incubated with 25 µg/mL of AarA S150A active site mutant (lower panels). The N-termini of individual species were inferred from their molecular mass, as indicated.

Figure S3. Protein sequences of the predicted substrates and non-substrates

All candidate substrates of AarA that we predicted in the *P .stuartii* genome were ranked according to their motif quality. The top fifteen candidate substrates and fifteen predicted non-substrates were selected and their TMDs and surrounding juxtamembrane regions were amplified from genomic DNA, *in vitro* translated and radiolabelled. Sequences of those protein fragments that were thus generated and tested for cleavage by AarA are shown aligned by the C-terminus of their predicted transmembrane domain (underlined). The number of amino acids that precede each tested fragment in the corresponding full-length protein are denoted by a number at the N-terminus of each sequence. The heptapeptide regions corresponding to the regular expression that encompasses the identified recognition motifs are highlighted in bold within each sequence and also listed separately in square brackets with individual motif quality scores indicated. Each protein is further identified by its NCBI accession and GI numbers, and its predicted topology. For example, 'o85-106i' indicates a periplasmic N-terminus, transmembrane domain spanning residues 85 to 106, and intracellular C-terminus; n10-21c29/30o signifies a signal peptide with a predicted signal peptidase cleavage site between amino acids 29 and 30. Proteins are listed in the order as they appear in Fig. 6C; those that were cleaved by AarA are marked with an asterisk.

Figure S3

Top-scoring candidate substrates:

*gi|183597385|ref|ZP_02958878.1||o3-28i|-----------------------------------1ES**TIATAAF**GSPWQLIIIALLIILIFGTKKLR…
['Motif:TIATAAFScore:13']

gi|183597515|ref|ZP_02959008.1||n12-25c30/31o186-211i|--166VQNH**RMQAAGV**IVDGDYHFNKYLLITLAIISIAIACIMGWFITLSITRPLGA…
['Motif:RMQAAGVScore:13']

*gi|183597857|ref|ZP_02959350.1||o688-708i|-----------------670GKGNKII**NITGAQA**ASGEDLLVWLLVLPAQASITLYFGKRKLRL…
['Motif:NITGAQAScore:13']

gi|183600335|ref|ZP_02961828.1||n10-21c29/30o158-182i|-------------151QDMAM**EIMVAQF**LPWLIALPIMLILFLWLLARALR…
['Motif:EIMVAQFScore:13']

*gi|183601034|ref|ZP_02962527.1||o18-39i|---------------------1SF**FISEAAA**SAGAPAQGNPYTMIIMLAVFALIFYFMILRPQQKR…
['Motif:FISEAAAScore:13']

gi|183597945|ref|ZP_02959438.1||o4-21i|---------------------------------------1**TWEYALI**GLVIGFIIGALVVRYGNPKLRQQKTAQA…
['Motif:TWEYALIScore:12']

gi|183596293|ref|ZP_02958321.1||o3-25i|--------------------------------------1D**IIILGVV**MFTLIVLVLTGLILFAKSKLVNTGNIKVEV…
['Motif:IIILGVVScore:11']

gi|188025396|ref|ZP_02997487.1||o10-33i|-----------------------------1**SVIYADF**SGFINFLNLLKILNWFLYLVCCFLIKNCDVFN
['Motif:SVIYADFScore:11']

*gi|188025653|ref|ZP_02959365.2||o156-183i|---------136KSGK**KLEIAEV**NLDDRRRELILQWFMYGGGVAGAGLIFGLILPHIIPRRKK…
['Motif:KLEIAEVScore:11']

gi|188025677|ref|ZP_02997623.1||o10-39i|----------------------0LLFTVNKFNGLSWF**LLFIALA**YCFGLIHSFTHLFCWLSFEHTKQQRQ
['Motif:LLFIALAScore:11']

*gi|188025732|ref|ZP_02959613.2||n10-21c29/30o85-106i|----------70LSAKSLL**TLSPAAI**EPLFFVFLSITALGIFYSNLFSRRAKR…
['Motif:TLSPAAIScore:11']

gi|188025749|ref|ZP_02959657.2||o13-42i|---------------------1**VYKESIM**N**TIRSSIV**V**LILLAII**TGVAYPLLVTGLANVLFP…
['Motif:VYKESIMScore:11','Motif:TIRSSIVScore:11','Motif:LILLAIIScore:13']

gi|183598643|ref|ZP_02960136.1||n16-35c40/41o54-74i|-------------40ATQVN**EMTLSFI**PKILSVIAVIIIAGPWMLNLLLDYMRTL…
['Motif:EMTLSFIScore:11']

Predicted non-substrates:

gi|188025349|ref|ZP_02997457.1||o16-36i|---------------------------------10SGRVSDKLTHFVFSLFITFMTLLSHGKDVVLK

gi|188025398|ref|ZP_02997489.1||o4-25i|------------------------------------0VVIENNLLPLSRVVVFCWLTVGILIDFDYNQMS…

gi|188025439|ref|ZP_02997503.1||o19-34i|----------------------------------12CSPSLVENGVYYIFILLTLLLERHIHLS

gi|183597229|ref|ZP_02958722.1||o4-19i|--0IQPLIWVRLLVRGRKAPAYRKRWGE…

gi|188025599|ref|ZP_02997581.1||o4-19i|--------------------------------------1QQLTASFYLFTLFCKFAIEKWFVRRQKFNL…

gi|188025656|ref|ZP_02997613.1||o4-24i|-------------------------------------0LSLEILWVVQNQSLVNVVFVFSLIKGKHLTIRFIYR…

gi|188025756|ref|ZP_02997655.1||o0-27i|-----------------------------------0LLSCHFLIFVIIFVVIYLSPQQIIMITDKVNHSLEK…

gi|188025787|ref|ZP_02997669.1||o18-36i|----------------------------------11ADFWRSDKNLIWLTLSSLLILKQITKFRPYEL

gi|188025931|ref|ZP_02997723.1||o4-29i|--------------------------------0LQYILQPILFFSVYHMMLLLIFACMYLVFKITDMKKI…

gi|188026116|ref|ZP_02997788.1||o10-31i|---------------------------------3SLYSPTWLYNNQNLILFPFINTYFFIINNSCSTNKM…

gi|183599599|ref|ZP_02961092.1||n4-14c21/22o231-247i|-----------------219AKIIATPFTVVADVVITPPLAIFVLIAFSK

gi|183601007|ref|ZP_02962500.1||o24-42i|-----------------------------11SGEMHEINVTPFIDVMLVLLIIFMVAAPLATVDIKVDLPAS…
